# Prevalence of hearing loss in college students: a meta-analysis

**DOI:** 10.3389/fnins.2023.1282829

**Published:** 2024-01-05

**Authors:** Myriam Kornisch, Ashley Barton, Hyejin Park, Rebecca Lowe, Toshikazu Ikuta

**Affiliations:** Department of Communication Sciences and Disorders, University of Mississippi, Oxford, MS, United States

**Keywords:** hearing loss, sensorineural hearing loss, noise-induced hearing loss, high frequency hearing loss, college students, university students, musicians, 6000 Hz

## Abstract

**Introduction:**

Hearing loss among college students, specifically noise-induced hearing loss (NIHL), appears to be increasing. This may be particularly challenging for this population as college students are required to listen to lectures in classrooms that may have suboptimal listening environments. College-aged musicians are at a particularly high risk due to repeated and extended exposure to loud noise. Therefore, the purpose of the current study was (1) to examine the prevalence of hearing loss in college students and (2) to emphasize the importance of detecting hearing loss at 6,000 Hz.

**Methods:**

A meta-analysis was conducted using the PRISMA model. The literature search yielded 8 studies (1,950 subjects) that tested hearing loss using an audiogram and Distortion Product Otoacoustic Emissions (DPOAEs). All studies used audiologic tests to detect hearing loss among college students between the ages of 17–35 years.

**Results:**

Results indicate that the prevalence of hearing loss in college students is 19%. In addition, the prevalence of hearing loss at 6,000 Hz is 85% among student musicians. For this meta-analysis, slight sensorineural hearing loss, or thresholds greater than 20 dB bilaterally or unilaterally, qualified as hearing loss.

**Discussion:**

Decreased hearing at 6,000 Hz may lead to an individual’s inability to hear important environmental factors and high frequency speech sounds. College students without full auditory function at this frequency may have difficulties performing in class based on decreased attention, comprehension, and memory. Although students may not realize the influence of their 6,000 Hz hearing loss or be unaware of its presence, it could significantly change their likelihood to succeed in college. Therefore, implementing a hearing conservation program may be advised for colleges and universities to help prevent hearing loss in students, particularly for collegiate musicians. In addition, it may be beneficial to screen hearing in college students at 6,000 Hz for better detection of hearing loss overall.

## Introduction

Although hearing loss is often associated with older adults, over 20% of individuals above the age of 12 in the United States (U.S.) are affected by auditory disorders (Cruickshanks et al., 1998; Goman and Lin, 2016). In fact, a potentially increasing number of young adults in the U.S. (i.e., individuals between 17and 35 years of age), particularly college students, present with some form of hearing loss (Rabinowitz et al., 2006; Shargorodsky et al., 2010). However, the reports of the prevalence of hearing loss in this population have been mixed in previous studies (Fulbright et al., 2017). In general, hearing loss may result from a variety of causes and is divided into three categories: (1) conductive loss (i.e., outer or middle ear impedance), (2) sensorineural loss, or (3) both (Isaacson and Vora, 2003). The focus of the current study will be sensorineural hearing loss, which is the term used when permanent damage occurs to the inner ear or auditory nerve. Sensorineural hearing loss can be caused by noisy recreational activities (e.g., hunting, attending concerts, playing loud music through headphones etc.), which might increase the risk for some college students to experience hearing-related symptoms, such as tinnitus, at a young age (Rawool and Colligon-Wayne, 2008; Phillips et al., 2010). Furthermore, high intensity sounds associated with certain recreational activities most often impact hearing at higher frequencies (e.g., 3,000 Hz, 4,000 Hz, 6,000 Hz) (Keppler et al., 2015a), which is thought to be the most common area for hearing loss in college students (Rabinowitz et al., 2006). These noisy activities may decrease hearing abilities at interoctave frequencies such as 6,000 Hz and, as a result, hearing loss could go undetected (Dempsey, 1985). Unfortunately, many college students are not properly educated about hearing loss or how to prevent it (DelGiacco et al., 2015; Berg et al., 2016). Therefore, they may be unknowingly increasing their likelihood of developing hearing disorders (Balanay and Kearney, 2015) by not using sufficient, if any, protection to prevent hearing loss (Rawool and Colligon-Wayne, 2008).

### Noise-induced sensorineural hearing loss

Noise-induced hearing loss (NIHL) is the leading cause of hearing loss in young adults (Martin et al., 2006). In general, NIHL is caused by exposure to loud noise, which can be defined as any noise over 85 dBA that someone is exposed to for more than 8 h (National Institute for Occupational Safety and Health, 1996). Temporary NIHL is caused by damage to the outer hair cells of the cochlea, and can occur within minutes of exposure to loud noise (Kujawa and Liberman, 2009). With extended exposure to loud noise, complete destruction of the outer and inner hair cells can occur, resulting in cell death (Kujawa and Liberman, 2009). As a result, NIHL typically forms a noise “notch” on an individual’s hearing abilities that is most commonly observed in the high frequencies (Phillips et al., 2010; Le Prell et al., 2011). More specifically, according to Coles et al. (2000), a noise notch is defined as a unilateral or bilateral decline in hearing at 3,000 Hz, 4,000 Hz, or 6,000 Hz that is 10 dB worse than the adjacent frequencies. However, despite the presence of a noise notch, NIHL can be difficult to diagnose due to its hidden progressive nature. Tests that examine auditory function, such as Distortion Product Otoacoustic Emissions (DPOAE), may be essential for detecting NIHL when it cannot be detected on an audiogram or other standard procedures (Henning and Bobholz, 2016). Therefore, when looking at the prevalence of hearing loss in college students, it is important to also look at early changes in the cochlea, as evident in DPOAEs. In addition to DPOAEs, interoctave frequencies that may not be evaluated in some procedures, such as 6,000 Hz, can be tested to detect primitive auditory changes, particularly in the high frequencies (Dempsey, 1985). Though the American Speech-Language-Hearing Association (ASHA) recommends evaluation protocols that include 6,000 Hz, this has not always been the case and may not be consistently followed in all settings (ASHA, 2005). This discrepancy between policy and practice may result in undetected hearing loss at this frequency. A lack of testing at 6,000 Hz may be because it is considered an interoctave frequency, meaning it might only be tested if there is a significant gap of 15 dB or greater between 4,000 Hz and 8,000 Hz (Dempsey, 1985). Furthermore, audiometric screening procedures currently recommend testing only 1,000 Hz, 2,000 Hz, and 4,000 Hz (ASHA, n.d.-a). Regardless of the type of testing used, early detection of hearing loss is important because it may significantly impact how students alter their own behaviors to prevent further hearing loss in the future (Henning and Bobholz, 2016).

In general, NIHL can subtly impact individuals’ ability to perceive and localize high-frequency sounds, including environmental sounds and some speech sounds. Accurate perception of environmental noises (e.g., bird chirping) aids in the ability to feel connected and increases an individual’s overall quality of life (Ramsdell, 1978; Shafiro et al., 2015; Moore, 2016). Likewise, if environmental sounds are diminished due to high frequency hearing loss, individuals may lose their security of a safe environment because they are unable to detect some hazardous sounds (e.g., traffic, sirens, alarms) (Gaver, 1993). High frequency hearing loss may also cause difficulty with localizing sound, which is one’s ability to recognize where a sound is coming from and how far away it is from the individual. Like the detection of environmental sounds, the ability to localize sound is important in emergency situations to determine where potential harm originates. In addition, this same sound localization is specifically useful for college students when they are listening to lectures and communicating at social gatherings since optimal high frequency hearing abilities are crucial for speech perception (Moore, 2016). For example, if an individual loses their hearing to any degree in the high frequencies, it could significantly impact their perception of/f/, /s/, and/θ/, as well as their discrimination capabilities of other speech sounds (ASHA, n.d.-c). As a result, these deficits in the ability to hear high frequency speech sounds may increase an individual’s cognitive effort needed to fulfill daily tasks (Ljung et al., 2009; Lewis et al., 2015), and deficits in perceiving high frequency sounds may result in a deficiency in the person’s potential to function efficiently and safely in all environments (Girard et al., 2009). This early detection of hearing loss at 6,000 Hz could be a valuable indicator of NIHL and may be helpful to bring awareness about prevention and protection that could offset any further impairment. For instance, detecting hearing loss at 6,000 Hz is an efficient way to prevent preliminary damage as the individual can alter their behavior before it progresses to the lower frequencies, which are more common in speech sounds (i.e., 500 Hz, 1,000 Hz, 2,000 Hz; Mehrparvar et al., 2018).

### Recreational activities and hearing loss

Overall, it is evident that NIHL can be detrimental to college students’ performance, and educating them about the harms of certain activities may help implement safer behaviors in the future (Jin et al., 2013; Keppler et al., 2015b). College students are at risk of developing NIHL as a result of recreational activities that often exceed recommended noise levels, such as (1) using personal listening devices, (2) hunting, or (3) attending concerts, bars, or sporting events (Balanay and Kearney, 2015). It should be noted that some recreational activities pose minimal risk (e.g., using a personal listening device at a low noise level), while others are considered high risk and could significantly impact hearing even with limited exposure (Henning and Bobholz, 2016). For example, sporting events are deemed high risk activities as they are associated with pain in the ear, hearing loss, and tinnitus, as well as excessive exposure to sounds above 85 dB (National Institute for Occupational Safety and Health, 1996; Balanay and Kearney, 2015). Nevertheless, the possibility of developing hearing loss during recreational activities may not concern students relative to the enjoyable aspects of the event, as the loud noise during these activities often increases enjoyment (Balanay and Kearney, 2015). Likewise, college students rarely use hearing protection during recreational activities due to discomfort, expense of equipment, and potential interference with the pleasure of the event or activity (Jin et al., 2013; Keppler et al., 2015b).

In addition to the damage caused by the loudness at recreational events and activities, the frequentness of these events and activities attended by college students can also pose a potential risk (Gupta et al., 2014). As mentioned previously, noise exposure can cause progressive damage and these frequent occurrences can amplify the development of further damage (Kujawa and Liberman, 2009). Because damage caused by recreational activities may appear temporary or non-existent early on, college students are often not aware of its progressive nature and how to form precautions related to noise exposure. Therefore, many students attend these loud events more than once a week for longer than the recommended limit (National Institute for Occupational Safety and Health, 1996; Balanay and Kearney, 2015). Not surprisingly, although most college students are unconcerned about the risk of damaging hearing during recreational activities, college students who have been affected by hearing loss or are aware of the potential negative impact of noise, tend to use more precautions (i.e., hearing protection use, decrease in noise exposure) when it comes to loud noise exposure (Balanay and Kearney, 2015; Keppler et al., 2015b). Because awareness increases preventative measures, conducting research that provides more information about the harm and influence of hearing loss and important precautionary behaviors to implement is crucial to increase hearing health among college students. This may ultimately aid in the establishment of hearing conservation programs, as well as optimize such program’s success (Jin et al., 2013; Keppler et al., 2015b).

### Hearing loss in the classroom

Regardless of the frequency at which hearing loss occurs, it is important to detect and treat hearing loss in college students, as noise interference in the classroom may negatively impact learning for any student (Dockrell and Shield, 2006). In fact, optimal hearing is crucial for success as college students must rely on the ability to hear in order to properly obtain knowledge throughout their college career (Lewis et al., 2015). Hearing loss may create challenges in a university setting due to the large classroom sizes and verbal lecture strategies (Dockrell and Shield, 2006; Rabinowitz et al., 2006). More specifically, classrooms often exceed the recommended noise level of 35 dBA, which can hinder any student’s ability to hear the speaker, especially when they present with impaired auditory functioning (ANSI, 2010; Lewis et al., 2015). Even if the hearing loss is mild (or hearing thresholds between 26 and 40 dB), factors such as excessive background noise, reverberation, and distance from the speaker could further impact a college student’s ability to accurately understand a spoken lecture (Dockrell and Shield, 2006; Larsen et al., 2008). Moreover, environmental noises in the classroom may interfere with a college student’s physical ability to comprehend, memorize, and apply what they hear (Ljung et al., 2009; Lewis et al., 2015), since these tasks require high cognitive effort and individuals with hearing disorders require even more effort to hear accurately and, consequently, experience auditory fatigue (Lewis et al., 2015).

In addition to hindering the learning process, hearing loss is often accompanied by other symptoms such as tinnitus, which is a sensation commonly associated with overexposure to loud noise that may impede daily functioning (Bhatt, 2018; Bramhall et al., 2018). This could potentially further affect a college student’s performance in an academic setting due to the hindrance it can have on sleep and an individual’s emotional state (Bhatt, 2018). As addressed previously, hearing conditions, such as tinnitus and high frequency hearing loss, that impact active listening in the classroom are often brought on by loud noise exposure (Phillips et al., 2010; Bramhall et al., 2018). Therefore, understanding not only the origin of hearing related problems in college students (e.g., noise exposure) but also the effect that these problems may have (e.g., on speech comprehension), can give more insight about how hearing loss influences learning and assist in developing preventive strategies to prevent further hearing damage. Due to the potential effect that hearing declines may have on college students, the current study aimed to (1) systematically gain a conclusive understanding of the prevalence of hearing loss in college students and (2) emphasize the importance of detecting hearing loss at 6,000 Hz.

## Methods

The current study was conducted as a meta-analysis using the Preferred Reporting Items for Systematic Reviews and Meta-Analyses guidelines (PRISMA; Moher et al., 2009).

### Data collection

**Figure 1:**
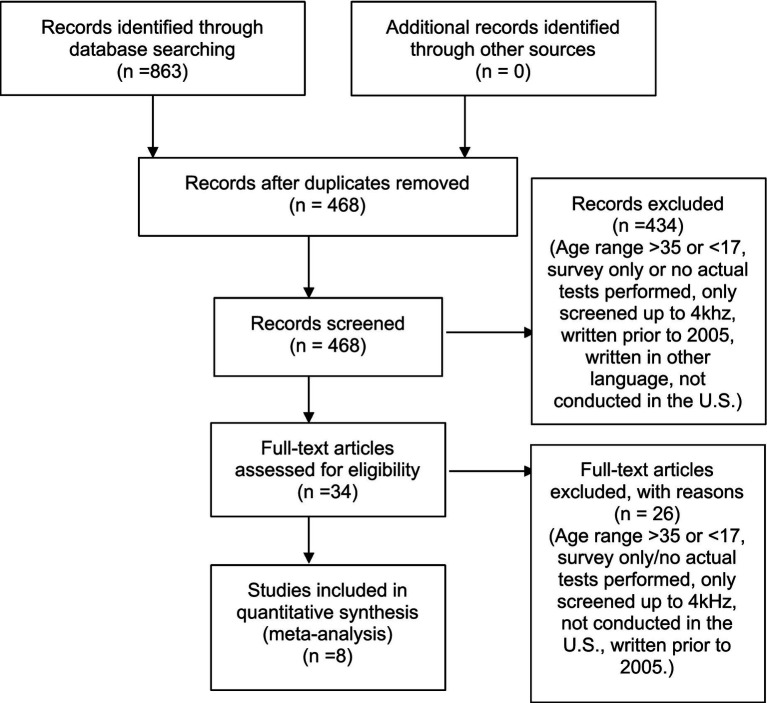
PRISMA flow diagram prevalence of hearing loss meta-analysis.

All articles were collected from the PubMed, ComDisDome, and CINAHL databases. The search terms (hearing loss) and (college students) were used in all databases. The term “college students” was chosen over “university students” because more articles used this term when conducting hearing loss studies. Figure 1 outlines the search process, including the reasons for exclusion.

### Inclusion criteria

Studies that included the following criteria were analyzed:

- Studies that exclusively included subjects who were enrolled in college. Studies that included young adults who were not enrolled in college or did not indicate if the young adults were enrolled in college were excluded.
- For the present study, young adults are defined as individuals between the ages of 17 and 35. Studies that included older college students (i.e., students above the age of 35) were excluded.
- To be included in data analysis, studies had to report true quantitative measures of auditory function, such as an audiogram, TEOAEs or DPOAEs. Studies that relied solely on qualitative and subjective measures (e.g., surveys) were excluded.
- Hearing screenings included a test of high frequencies of at least 6,000 Hz.
- Only studies written in English were included in analysis.
- Only studies conducted in the United States were included in analysis.
- Studies were published in peer-reviewed journals.

### Exclusion criteria

- Studies that were published before January 1, 2005, were excluded.
- Studies that included subjects with documented congenital hearing loss or hearing loss because of disease or genetics were excluded.
- Studies that included participants with a middle ear pathology, indicated by Tympanometry measures that were Type B or Type C were not included in the study.
- Studies that included participants with a middle ear pathology, as indicated by an occluded ear canal observed by otoscope, were not included in this study.

### Screening and coding

The first step in the screening process was to remove all duplicate articles. Remaining articles were screened based on their titles and abstracts. Articles that did not fit the inclusion or exclusion criteria were disregarded. Articles that appeared to meet the inclusion and exclusion criteria or those that did not provide enough information to determine were further screened. All remaining articles were examined in their entirety and included or excluded based on all criteria listed above. All studies included in the final meta-analysis tested hearing loss using quantitative measures (i.e., threshold levels, notched audiograms, or present and absent DPOAEs). Audiologic tests were selected over self-report measures due to the unreliability of self-reported hearing loss (Widén et al., 2009). When analyzing the articles that measured hearing loss as elevated threshold levels, we considered hearing loss to be bilateral or unilateral thresholds >20 dB, which is considered a slight hearing loss at any frequency within the range of human hearing (ASHA, n.d.-b). Although adults are typically screened at 25 dB, college students likely experience similar settings as school children, who are typically screened at 20 dB, such as reverberation, distance from the teacher, etc. that require more sensitive hearing. Therefore, it is important to detect slight hearing loss (Dockrell and Shield, 2006; Larsen et al., 2008). As outlined by Coles et al. (2000), notched audiograms had bilateral or unilateral thresholds at least 10 dB worse at 3,000 Hz, 4,000 Hz or 6,000 Hz than at 1,000 Hz, 2,000 Hz, and 8,000 Hz. One study utilized DPOAEs, which are measured as absent or present (Henning and Bobholz, 2016). Absent DPOAEs indicate hearing loss. Furthermore, another study utilized both audiometry and TEOAEs to detect hearing loss (Jin et al., 2013). Regarding the obtainment period for data, the initial search was for any articles published after July 1, 2005; however, the publication range for all articles included in this meta-analysis was between 2008 and 2020. As for as the individual articles, they do not state the time periods in which they recruited participants or collected data.

### Hearing loss variables included in data analysis

In some studies, such as Le Prell et al. (2011) and Phillips et al. (2010), hearing loss was reported using multiple measures, such as decreased thresholds and notched audiograms. For these studies, only one measure was chosen for the meta-analysis. The measure chosen for the Le Prell et al. (2011) article was based on which information was most clearly reported. Le Prell et al. (2011) indicated the number of notched audiograms as hearing loss per subject but indicated results of decreased thresholds as hearing loss per ear. For example, they noted that four subjects had notched audiograms but said that eight ears had hearing loss greater than 25 dB. Results of the notched audiograms were chosen because it was clear the number of subjects who had hearing loss. For the decreased thresholds, it was not clear if the ears represented one person or whether both ears were included for some subjects. As a result, there were more ears with hearing loss than subjects with hearing loss. For the (Phillips et al., 2010) study, we chose the subjects with notched audiograms over the subjects with sloping thresholds. This was done because the purpose of that study was to find the prevalence of students with noise-induced hearing loss, and the researchers only indicated hearing loss in subjects with notched audiograms in their further analysis. In two studies (Phillips et al., 2008; Jin et al., 2013), hearing loss was reported for multiple years, with some new subjects in the subsequent years and some repeat subjects. In their findings, it was not clear which results were from the new subjects and which ones were from the returning subjects. For the present study, analysis focused on the first-year results only to ensure that there was no overlap of participants skewing results. The decision to focus on thresholds at 6,000 Hz was based on the tendency of noise notches being observed at this frequency for musicians. For example, Fearn (1993) assessed thresholds of 220 student musicians between the ages of 16 and 30, and it was reported that 75% of elevated thresholds were seen at the 6,000 Hz frequency. Additionally, Jansen et al. (2009) saw noise notches to most occur at the 6,000 Hz frequency when investigating the hearing of 241 musicians between the ages of 23 and 64. These articles provide support for 6,000 Hz being a useful frequency for detection of noise notches, and thus, they played a part in the decision to select this frequency. As for how a slight sensorineural hearing loss was defined in this study, the criteria used for degrees of hearing loss in this meta-analysis were those presented by Clark (1981) and accepted by ASHA, which designates slight hearing loss as falling within 16–25 dB (ASHA, n.d.-b). As for the definition for sensorineural hearing loss, this meta-analysis abided by the standard definition set out by Isaacson and Vora (2003), which describes it as a hearing loss due to an issue within the inner ear. Thus, according to those definitions, a slight sensorineural hearing loss is a hearing loss with a degree that falls within 16–25 dB and is due to a problem in the inner ear.

### Meta-analytic procedures

A meta-analysis was conducted with the meta-R package (Balduzzi et al., 2019). A random intercept logistic regression model was used to estimate overall proportion (Stijnen et al., 2010). Given considerable variance in the measurements of hearing, overall proportions were computed using a random intercept logistic regression model (Stijnen et al., 2010) and a random effects model (Borenstein et al., 2009), as well as fixed effects model. Between-study variance τ^2^ was calculated with Maximum-likelihood estimator (Viechtbauer, 2005). Confidence interval of 95% was estimated to determine the reliability of the overall proportion. Additional tests were conducted to estimate the potential influences of publication bias. To detect funnel plot asymmetry, Egger’s regression test was used (Egger et al., 1997). Unpublished studies were estimated by the trim and fill method (estimation of unpublished studies) (Duval and Tweedie, 2000).

## Results

A total of 863 articles were in the initial literature search. After removing duplicates, 468 articles remained. After screening abstracts and titles, an additional 434 articles were excluded for not meeting inclusion and exclusion criteria. The remaining 34 articles were screened through a full-text review as it could not be determined if they met criteria based on their titles or abstracts alone. From this full-text review, 26 additional articles were removed. In total, 8 studies (1,950 subjects) remained that met inclusion and exclusion criteria. These studies were analyzed in the full meta-analysis. A summary of the included studies’ characteristics can be seen in Table 1.

**Table 1:** Summary of included studies’ characteristics.

First author	Year	Sample size	Ex. group size	Assessment	Frequencies assessed
Alessio et al.	2020	182	182	Air conduction pure tone threshold	250–8,000 Hz
Gopal et al.	2013	25	14	Air conduction pure tone threshold	250–8,000 Hz
Henning and Bobholz	2016	63	28	Air conduction pure tone threshold & DPOAE	500–8,000 Hz
Jin et al.	2013	698	350	Air conduction & bone conduction pure tone threshold	250–8,000 Hz
Le Prell et al.	2011	56	56	Presence of noise notch	500–8,000 Hz
Phillips et al.	2008	110	110	Air conduction pure tone threshold	250–8,000 Hz
Phillips et al.	2010	329	329	Presence of noise notch	1,000–8,000 Hz
Widén et al.	2009	258	258	Air conduction pure tone threshold	500–6,000 Hz

### Meta-analysis

**Figure 2:**
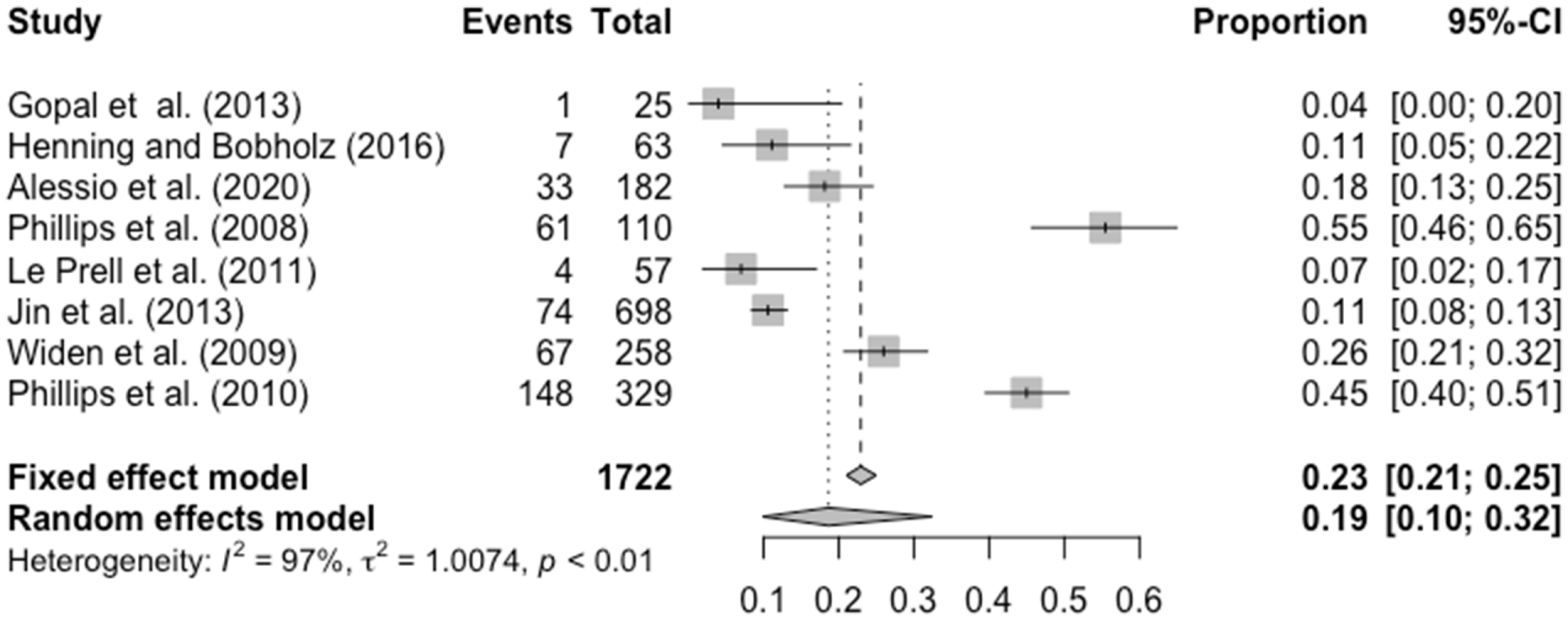
Forest plot for the prevalence of hearing loss in college students including all studies.

The present meta-analysis examined prevalence of hearing loss in college students by (1) examining the total number of participants in each study and (2) the number of participants with hearing loss. In addition, the random effects model was used due to the variation of measures used to assess hearing loss. Heterogeneity for prevalence of hearing loss was as follows, *τ*^2^ = 1.00, *I*^2^ = 97% and *p* < 0.01. The prevalence of hearing loss in college students in the United States, as estimated from the current meta-analyses, ranged from 4 to 45% with the overall prevalence among the studies being 19% (95% CI = 0.10; 0.32). The results for prevalence of hearing loss are displayed in Figure 2.

Rank correlation test did not indicate funnel plot asymmetry. However, trim and fill discovered two missing studies in favor of more prevalence. Adding these two missing studies would increase estimated overall prevalence to 25% (95% CI = 0.14; 0.42).

### Subgroup meta-analysis

**Figure 3:**
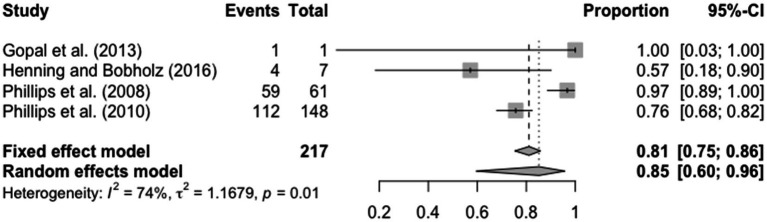
Forest plot for prevalence of 6,000 Hz hearing loss in college students including 4 studies.

For the prevalence of 6,000 Hz loss among students with a hearing loss, the (1) total number of participants with hearing loss and (2) number of participants with a loss at 6,000 Hz were used to look at what percentage of students with hearing loss had a loss at 6,000 Hz. For this component, heterogeneity was as follows, *τ*^2^ = 1.17, *I*^2^ = 74% and *p* = 0.01. The prevalence of students with a 6,000 Hz loss among the overall students who have hearing loss in each study ranged from 57 to 100%, with the average prevalence of hearing loss across the four studies being 85% (95% CI = 0.60; 0.96). The results for the prevalence of hearing loss at 6,000 Hz are displayed in Figure 3.

## Discussion

The present meta-analysis combined the results of previous studies to look at (1) the prevalence of hearing loss in college students (Jin et al., 2013; Keppler et al., 2015b) and (2) emphasize the importance of detecting hearing loss at 6,000 Hz (Phillips et al., 2008, 2010; Gopal et al., 2013; Henning and Bobholz, 2016). First, we determined the prevalence of hearing loss in college students. This was done to gain a better understanding of the influence of hearing loss on college students, and to increase awareness of the importance of hearing health in this population since knowing the magnitude of the impact of hearing loss may lead to safer hearing behaviors in the future (Balanay and Kearney, 2015; Keppler et al., 2015b). Results indicate that the prevalence of hearing loss in college students is 19%. This finding is much lower than the 55% of hearing loss found in college students as reported by Phillips et al. (2008). The difference in prevalence of hearing loss may be due to the difference in populations observed. Phillips et al. (2008) solely observed hearing performance in music students, while this meta-analysis included students pursuing various academic careers. Music students, such as those observed in the Phillips et al. (2008) study, are often exposed to loud noise that exceeds the recommended limit and, therefore, may be more susceptible than other students to developing declines in hearing (Gopal et al., 2013). Although not all college students are exposed to loud noise as frequently as music students, most young adults should not experience hearing declines due to aging. Therefore, the majority of the hearing loss detected in this meta-analysis is assumed to be acquired due to some form of noise exposure, which has previously been considered the most common form of hearing loss in young adults (Cruickshanks et al., 1998; Martin et al., 2006). In general, hearing disorders in college students are often preventable if appropriate precautions are used. Consequently, more research needs to be done to better understand which specific student populations (e.g., music majors) may be most influenced by noise-induced hearing loss, as well as to identify ways to decrease the development of hearing disorders. Previous studies have introduced the idea of implementing programs designed to educate students about the risk of hearing loss and ways to prevent it (Jin et al., 2013; Keppler et al., 2015b). The results of this meta-analysis agree with these suggestions and support the claim of a general need for the implementation of preventative programs.

Another important component of optimizing hearing health is early detection, which can be established by testing interoctave frequencies (Dempsey, 1985). In the current study, we found that among students with hearing loss, 85% presented with a loss at 6,000 Hz. This is a high percentage of students who are impacted by a hearing loss greater than 6,000 Hz. In a previous study that looked at the prevalence of hearing loss in college students, they did not observe 6,000 Hz and the researchers mentioned that it may have affected the outcome (Alessio et al., 2020). In contrast, Henning and Bobholz (2016) did record hearing changes in college students at 6,000 Hz but found the prevalence to be 57% which is less than what was found in this study. The difference in results from Henning and Bobholz, as compared to the current study, may be due to the difference in testing. For this meta-analysis, both DPOAEs and audiograms were used to determine the prevalence of hearing loss at 6,000 Hz. However, Henning and Bobholz only used DPOAEs, which objectively test auditory function by examining the activity of outer hair cells in the cochlear (Henning and Bobholz, 2016). Alternatively, audiograms rely on response from an individual, which may lead to false results (Jin et al., 2013). Therefore, DPOAEs may yield more reliable results based on the objective nature of the test as compared to the audiogram, which relies on an individual’s response. Although the two studies show varying degrees of prevalence, hearing loss at 6,000 Hz is significant in both studies. Therefore, based on the high percentage of students with a hearing disability, who had a loss at 6,000 Hz in this meta-analysis, it is important to consider testing 6,000 Hz in all hearing evaluations and screenings for college students. Testing for hearing loss at 6,000 Hz could lead to earlier detection of hearing loss and, consequently, quicker implementation of intervention and education for the student (Dempsey, 1985).

### Implications

One implication of this meta-analysis is the importance of developing hearing conservation programs for university students, particularly student musicians (Jin et al., 2013; Balanay and Kearney, 2015). Educating students on the (1) risk of hearing loss, (2) importance of hearing protection, and (3) danger of noisy activities could lead to more precautions on their part that may help decrease the prevalence of hearing loss (Balanay and Kearney, 2015; Keppler et al., 2015b). Balanay and Kearney (2015) found that, although most college students are attending noisy activities (i.e., concerts, bars, sporting events), they are unlikely to use hearing protection unless they have already experienced auditory changes. Upon further examination, they found that college students with hearing loss are overall more likely to implement safe hearing behaviors into their daily life. This indicates that knowledge of hearing loss could lead to using precautions, which implies that education about hearing health may be successful (Balanay and Kearney, 2015; Keppler et al., 2015b). One useful strategy included in hearing conservation programs is to use simulations that mimic auditory disorders, such as tinnitus or decreased thresholds, as these simulations can boost understanding of the importance of protecting hearing health (Balanay and Kearney, 2015). College students who have not experienced changes in their hearing may be unaware of the effects of risky activities, such as attending concerts and bars or listening to loud music and, as a result, may not be inclined to alter their behaviors. Thus, education and simulations may assist to increase awareness (Rawool and Colligon-Wayne, 2008; Balanay and Kearney, 2015). However, for hearing conservation programs to be successful, it is important to incorporate them at an ideal time. At most universities, students are required to attend an orientation or some form of college experience/health class before they begin classes in the fall or during their first semester. During orientation, students are educated about important collegiate experiences and risks, such as how to succeed in classes, getting along with roommates, alcohol safety, etc. Similar topics are addressed in introductory college experience/health classes. Hearing conservation training could easily be implemented during orientation or introductory classes to ensure that students are practicing hearing safety along with other school protocols that can lead to optimal success (Balanay and Kearney, 2015). Even if not implemented at orientation or in introductory classes, the sooner and more widespread the education is, the more effective it will be long-term (Jin et al., 2013; Balanay and Kearney, 2015; Keppler et al., 2015b). For example, knowledge about hearing loss can lead to using preventative measures that decrease the development of hearing disorders. Thus, utilizing conservation programs early on in a college student’s career could be a useful tool to implement these changes.

Another major implication of this study is to understand the importance of screening individuals, especially college students, at 6,000 Hz. Currently, standard hearing screening procedures do not routinely include 6,000 Hz. As a result, hearing loss may go unnoticed in some students (e.g., college musicians). This decreased hearing at 6,000 Hz may lead to an individual’s inability to hear important environmental factors, as well high frequency speech sounds (Moore, 2016). Furthermore, college students without full auditory function at this frequency may have difficulties performing in class based on decreased attention, comprehension, and memory (Girard et al., 2009; Ljung et al., 2009; Lewis et al., 2015). Specific challenges that hearing loss may pose in the classroom include difficulty hearing content and following instructions, particularly for words that include the sounds at the frequency of which the student has a hearing loss. For students who already have a disability, such as a hearing loss, listening against competing stimuli may be more difficult (Dockrell and Shield, 2006). Even when students hear what is being said and can repeat it, those with mild to moderate hearing loss may have difficulty with comprehending more complex language (Lewis et al., 2015). Although students may not realize the influence of their 6,000 Hz hearing loss or be unaware of its presence, it could significantly change their likelihood to succeed in college (Dockrell and Shield, 2006; Larsen et al., 2008). Early detection of high frequency hearing loss may also prevent further NIHL affecting lower frequencies in the future (Mehrparvar et al., 2018). Therefore, to create awareness about auditory changes at 6,000 Hz and possible ways to prevent it, it is crucial to be aware of its impact and occurrence. Hence, understanding the prevalence of 6,000 Hz hearing loss among college students with hearing loss is important in determining the need of routinely screening students at this frequency. If a high percentage of students with hearing loss are affected at 6,000 Hz, then that indicates that this loss is significant and should be screened. Hearing loss at 6,000 Hz is frequently an early indication of noise-induced hearing loss and may be the only impairment an individual experiences. Routine hearing screenings, provided by university health centers or clinics throughout the year, may encourage students to have their hearing screened. Thus, identifying more students who have a hearing loss, particularly at 6,000 Hz. University health centers already provide free services to students, and screenings advertised and administered on campus in convenient locations may increase accessibility and interest in student’s having their hearing screened. If students fail the screening, they can then be referred to the university health center or hearing clinic for further evaluation and to receive services as necessary. Without screening of this frequency, early detection of hearing disorder for a student with impairment solely at this frequency may be absent (Dempsey, 1985).

A third implication of this meta-analysis is the importance of targeted interventions implemented by universities to support students with hearing loss. Hearing loss can impact both educational success and students’ future success in occupational settings. As college is in preparation for a student’s career, academic success in college related to hearing loss will influence students’ future occupations. Additionally, hearing loss in adults has been related to higher rates of unemployment (Shan et al., 2020). For student musicians, hearing loss may impact their current or future practice if they decide to pursue professional music (Henning and Bobholz, 2016). Therefore, the importance of supports for students who already have hearing loss cannot be understated. Previous studies have found that many classrooms do not meet the American National Standards Institute (ANSI) protocols for classroom acoustics needed for optimal hearing (Larsen et al., 2008; Lewis et al., 2015). Better classroom acoustics that are up to standards would improve classroom hearing abilities for all students, including those with hearing loss. With the identification of students who have an undetected hearing loss, student support services can be provided such as preferential classroom seating or speaker amplification. For student musicians, ear plugs could be provided by the university for at school practices and performances to help in the prevention of further hearing damage (Jin et al., 2013). Some of these suggestions could easily be implemented by universities not only to serve students with hearing loss but to also prevent further hearing loss in the future.

### Limitations

The most significant limitation of the current study is that only articles including students pursing a degree in music were included in the analysis of prevalence of hearing loss at 6,000 Hz. Other students pursuing a variety of degree paths are included in the overall prevalence, but the results of this meta-analysis may over-represent musical students in the general population of college students. Nevertheless, young adults are frequently exposed to loud music and noisy recreational activities regardless of their degree path. Another limitation of the current study is that for two of the articles (Jin et al., 2013) and (Phillips et al., 2008), results were reported for multiple years. We decided to only report results of 1 year to prevent overlap. That being said, for Jin et al. (2013), results for the same students changed over the years. They suggest that findings from the first year, which we included in this meta-analysis, could have been due to false negative results and should be considered with caution (Jin et al., 2013). Phillips et al. (2008) and Phillips et al. (2010) were collected in different years and therefore they were independently sampled. However, this does not fully exclude the possibility that some of the same individuals participated in these two studies. Another limitation of this study is that the articles included tested hearing loss using different measures. This led to a high heterogeneity score for the meta-analysis. Lastly, for the subcategory of prevalence of hearing loss at 6,000 Hz, only a few articles presented data. This limited the sample and, therefore, may not be representative of a larger population. Further, hidden hearing loss is a recently recognized disorder in which standard audiometric tests fail to detect hearing loss caused by cochlear neuropathy (Grinn and Le Prell, 2022). As a result, the prevalence of hearing loss at 6,000 Hz could potentially be higher due to hidden hearing loss. Additionally, another limitation is the potential source of bias present in the selection of participants. Specifically, by recruiting college students that are musicians or music majors, there may be missing data for other students that may be exposed to loud noise. For example, engineering students may be exposed to loud machinery, and theater and film major may also have to work with loud sound effects or sound editing. A further limitation could lie in the slight heterogeneity present within the age range selected. The large gap between 17-year-old subjects and 35-year-old subjects could influence the results. Specifically, the difference in hearing loss could be different for a 17-year-old compared to a 35-year-old due to the amount of exposure they have had. For example, a 35-year-old Ph.D. student for music will have had a much longer exposure to music than a freshmen music major. Furthermore, the generalizability of the findings is another limitation due to a singular frequency being evaluated. Although 6,000 Hz has been shown to be a frequency of interest in previous studies, this meta-analysis did not consider other frequencies in the 3,000–6,000 Hz range that is typically noted as presenting noise notches. Having a closer look at noise notches present in lower frequencies could provide a better understanding as to the effects of noise on everyday hearing, which may occur at lower frequencies. Finally, not all studies included in this meta-analysis described steps taken to ensure their assessment instruments were calibrated. This has the potential of introducing error to the results, especially for audiometric testing.

### Future directions

Based on the results from this study, we found that hearing loss is significantly impacting the college student population, particularly student musicians. The most common form of hearing loss among college students was found in the high frequencies, which indicates noise-induced hearing loss (Phillips et al., 2010). Because of the known effects that noise can have on hearing, it may be important to look at college student groups that may be at an increased risk of hearing loss based on their loud noise exposure (Martin et al., 2006). One group that may be at an increased risk are students pursuing music-related careers. These students are often in orchestras or marching bands with several instruments playing simultaneously. When multiple instruments are playing together, the noise increases significantly. On an average week, music students are exposed not only to levels above 85 dBA but also for longer than the recommended limit (Gopal et al., 2013). Due to the increased noise levels that music students are exposed to daily, it may be useful to conduct future research that looks at the potential heightened risk of hearing loss in this specific population.

Additionally, we have suggested the importance of implementing hearing loss conservation programs in university settings to increase student awareness on the impacts of NIHL. However, information on the effectiveness of programs of this type has not yet been studied. While students’ knowledge of the effects of NIHL appears to be correlated with the use of safer hearing habits, many of these students already have hearing loss (Balanay and Kearney, 2015; Keppler et al., 2015b). Therefore, the effectiveness of educational programs in preventing NIHL is unknown. There may also be class differences in knowledge of NIHL and protective practices (Berg et al., 2016). According to Berg et al. (2016), freshman may be less knowledgeable and, therefore, more likely to engage in noisy activities that can contribute to NIHL, emphasizing the value of implementing hearing conservation programs for incoming college students. Future research may look at the effectiveness of hearing conservation programs in universities, particularly between classes, to determine the best way to present hearing loss education to college students.

### Concluding remarks

Gaining a better understanding of the impact that hearing loss is having on college students may lead to more awareness about the importance of educating college students about (1) hearing loss in general and (2) hearing protection specifically. In addition, the frequencies impacted seem to play a crucial role in this specific population with hearing loss. Based on the current study, it appears that (1) the overall prevalence of hearing loss in college students is 19% and (2) 85% of collegiate musicians experience hearing loss at 6,000 Hz. Thus, we agree with previous research suggesting that implementing hearing conservation programs early on in a student’s college career would be beneficial to diminish the prevalence of acquired hearing loss in college students. Moreover, due to the high percentage of college students who have a hearing loss that were affected at 6,000 Hz, this study emphasizes the importance of routinely testing 6,000 Hz in standard audiologic screenings for this population. Lastly, we have stated the importance of implementing hearing loss conservation programs in university settings, as well as universities providing supports for college students who have a hearing loss.

## Data availability statement

The original contributions presented in the study are included in the article/supplementary material, further inquiries can be directed to the corresponding author.

## Author contributions

MK: Conceptualization, Methodology, Supervision, Writing – original draft, Writing – review & editing. AB: Data curation, Writing – original draft. HP: Writing – review & editing. RL: Writing – review & editing. TL: Writing – review & editing, Formal analysis, Methodology, Visualization.
